# Motivational orientation and practice-scheduling patterns as correlates of self-regulated learning strategies in pre-professional cello students: a predictive-correlational study

**DOI:** 10.3389/fpsyg.2026.1929562

**Published:** 2026-08-31

**Authors:** Şehriban Koca, Berrin Melisa Altunoluk

**Affiliations:** 1 Department of Musicology, Faculty of Music and Performing Arts, Mersin University, Mersin, Türkiye; 2 Department of Music Education, Institute of Educational Sciences, Mersin University, Mersin, Türkiye

**Keywords:** cello education, comprehension monitoring, learning strategies, practice scheduling, self-regulated learning, self-reported motivational orientation

## Abstract

**Background:**

Self-regulated learning (SRL) theory and Self-Determination Theory (SDT) propose that motivational orientation is statistically linked to the cognitive and metacognitive strategies learners use during independent practice. Prior research on instrumental practice—including earlier descriptive analyses of this population—has relied on concurrent correlational designs that cannot establish predictive ordering or test underlying processes.

**Aim:**

This predictive-correlational study examined whether a self-reported, single-item indicator of primary motivational reason *(interest/mastery-oriented vs. exam-oriented)* and a self-reported practice-scheduling pattern (daily/structured, unstructured, or pre-examination) were statistically associated with five strategic practice dimensions—attention, rehearsal, elaboration, organization, and comprehension monitoring—and whether comprehension monitoring statistically mediates the association between these variables and academic achievement and self-perceived competence.

**Methods:**

Participants were 166 pre-professional cello students from Fine Arts High Schools in Türkiye (convenience/purposive sample; school identifiers were not retained, precluding multilevel analysis). Data were analyzed using Welch’s *t*-tests and Welch’s ANOVA with Games–Howell *post-hoc* comparisons, hierarchical multiple regression with full diagnostic checks (including HC3-robust inference for one heteroscedastic model), and bootstrapped (10,400 resamples) mediation analysis; because the proportionality assumption of the ordinal logistic model was violated, mediation was re-estimated using multinomial logistic regression, which does not require this assumption. Holm-Bonferroni correction was applied within each hypothesis’s family of five strategy-dimension tests.

**Results:**

Self-reported interest/mastery-oriented motivation was associated with higher rehearsal, elaboration, and comprehension-monitoring scores. The hypothesized (H3) block-level associations of motivation and scheduling with elaboration and comprehension-monitoring strategy use did not survive Holm correction; an unhypothesized, exploratory association between motivation and rehearsal was the only regression finding to survive correction (Δ*R*^2^ = 0.107, *p*_Holm < 0.002) and is reported as a hypothesis-generating finding rather than a confirmed one. Self-reported daily scheduling was associated with higher comprehension-monitoring and attention scores. Under the assumption-appropriate multinomial specification, none of the twelve bootstrapped indirect effects (motivation and both scheduling contrasts, via comprehension monitoring, on achievement and competence) reached statistical significance.

**Conclusion:**

Self-reported motivational orientation is statistically associated with several, but not all, self-regulated practice strategies in this sample, with the strongest and most robust association observed for an unhypothesized dimension (rehearsal); comprehension monitoring did not emerge as a statistically supported mediating mechanism once model assumptions were satisfied. Findings are correlational, based on single-item self-report measures and a non-probability sample from one country, and should be interpreted as hypothesis-generating rather than confirmatory.

## 1 Introduction

### 1.1 Musical instrument learning as a self-regulated learning process

Learning to play a musical instrument is widely recognized in the educational psychology literature as a paradigmatic context for studying self-regulated learning (SRL), because it requires learners to independently set performance goals, select and monitor strategies during practice, and evaluate outcomes with only intermittent external feedback from a teacher (McPherson and Zimmerman, 2011; Zimmerman, 2002). Zimmerman’s (2002) cyclical SRL model frames this process across three interdependent phases—*forethought* (goal-setting and strategic planning), *performance* (strategy enactment and self-monitoring), and *self-reflection* (self-evaluation and strategy adjustment). Winne and Hadwin’s (1998) information-processing model of SRL offers a complementary account, describing learning as a recursive sequence of task definition, goal-setting and planning, strategy enactment, and adaptation, in which the motivational and behavioral conditions a learner brings to a task are theorized to shape the cognitive and metacognitive operations subsequently deployed.

For a fretless string instrument such as the cello, where accurate intonation depends on continuous self-generated auditory feedback rather than fixed physical reference points, the demands placed on a learner’s self-regulatory capacity are particularly high (Hallam, 1997; McPherson and Renwick, 2001). Within educational psychology, this kind of error-detection-and-correction behavior is understood as an instantiation of metacognitive monitoring—the learner’s ongoing awareness and evaluation of their own cognitive activity during task performance (Flavell, 1979; Pintrich et al., 2000; Schraw, 1998). Instrumental practice therefore provides a well-suited applied setting in which to examine how learners’ self-reported motivational orientation and practice-scheduling patterns are statistically associated with concrete, self-reported strategic actions (Hemmler and Ifenthaler, 2024; Panadero, 2017).

A recurring theoretical claim across these SRL models is that motivation functions as a proximal correlate, and plausibly a directional influence, on strategic behavior: the reasons learners report for practicing tend to co-occur with how they report practicing (Pintrich, 2000; Zimmerman, 2002). We note explicitly that the present, cross-sectional design cannot adjudicate between motivation shaping strategy use, strategy use shaping motivation, or both being jointly produced by an unmeasured third variable; directional language used elsewhere in this manuscript reflects the *a priori* theoretical ordering specified by these models and should be read as a hypothesis under test, not as an established causal fact. Much of the instrumental-practice literature—including prior descriptive analyses of the population examined in this study—has tested this claim through concurrent correlational designs that establish covariation between motivational and strategic variables without specifying a directional, predictive ordering. This leaves an important theoretical proposition under-tested in this population: whether self-reported motivational orientation is a statistically robust correlate of the specific learning strategies students report using.

### 1.2 Motivational orientation as a correlate of strategic behavior

Self-Determination Theory (SDT; Deci and Ryan, 2000; Ryan and Deci, 2020) distinguishes autonomous (intrinsic) motivation—engagement driven by inherent interest and a genuine desire to master a task—from controlled (extrinsic) motivation, in which engagement is driven by external contingencies such as grades or evaluative pressure. Autonomous motivation has been linked to deeper information processing and greater reliance on elaboration and organizational strategies, whereas controlled motivation is more consistently associated with surface-level, repetition-based strategy use (Ryan and Deci, 2000; Vansteenkiste et al., 2006). Achievement goal theory offers a complementary account: learners pursuing mastery goals tend to report deeper, more metacognitively demanding behavior than learners oriented toward performance-avoidance goals (Elliot and McGregor, 2001; Pintrich, 2000). In the specific context of instrumental practice, intrinsic motivation has been identified as associated with persistence and with the use of sophisticated metacognitive strategies such as comprehension monitoring and error correction (Evans, 2015; MacIntyre and Potter, 2014; Seifert, 2004; Zimmerman, 2002).

### 1.3 Practice-scheduling patterns and deliberate practice as behavioral correlates of self-regulation

A second, conceptually distinct correlate of strategic behavior is the self-reported pattern of practice scheduling—whether a learner distributes practice across time in a structured, anticipatory manner or concentrates it reactively before an evaluation. Scheduling regularity is one of the core forethought-phase processes in Zimmerman’s (2002) cyclical model and has been linked in the instrumental-practice literature to more systematic error identification and correction (Hallam, 2001; Nielsen, 1999). Distributed practice is theorized to create the temporal conditions for monitoring-and-adjustment cycles to unfold, whereas massed or pre-examination practice is theorized to compress this cycle (Bonneville-Roussy and Bouffard, 2015; Ericsson et al., 1993).

This temporal dimension is related to, but conceptually distinct from, the qualitative dimension captured by deliberate practice: effortful, goal-directed engagement performed at the edge of a learner’s current competence (Ericsson et al., 1993). The expertise-development literature identifies deliberate, strategically managed practice—not raw practice quantity—as a key differentiator between advanced and less advanced performers (Hallam, 2001; Sloboda et al., 1996). From an educational-psychology standpoint, this quality dimension is consistent with cognitive load theory (Sweller, 1988), which holds that learners have a limited capacity for actively processing new information during a task; conditions that reduce unnecessary processing demands may free capacity for higher-order strategic activity such as elaboration, error diagnosis, and self-monitoring (Muelas and Navarro, 2015). Techniques such as slow practice (Allingham and Wöllner, 2022; Maxfield, 2018) and segmenting a piece into smaller practice units (Nielsen, 1999) exemplify strategic behaviors through which students may regulate this processing load.

### 1.4 Strategic practice behavior: a five-dimensional taxonomy

Instrumental practice research has documented a taxonomy of strategic behaviors consistent with general models of learning strategies (Weinstein and Mayer, 1986): attentional strategies, rehearsal strategies, elaboration strategies, organizational strategies, and comprehension-monitoring strategies (Aydıner Uygun and Kılınçer, 2017; McPherson and Renwick, 2001; Nielsen, 1999). While descriptive work—including prior analyses of the present sample—has established the relative frequency with which cello students report using each strategy type, it has not formally tested whether self-reported motivational orientation and practice-scheduling patterns are statistically associated with each strategy dimension once practice quantity and contextual variables are held constant, nor whether such associations survive correction for multiple comparisons.

### 1.5 Integrating the framework

Synthesizing these strands, the present study situates strategic practice behavior as the point of intersection between two theoretically distinct but empirically entangled self-reported correlates—motivational orientation and practice-scheduling pattern—net of the quantity of practice performed. This synthesis is consistent with cyclical SRL models (Winne and Hadwin, 1998; Zimmerman, 2002) and extends this logic to pre-professional cello students, in whom strategic self-regulation operates under the elevated processing demands described by the cognitive-load framework above, owing to the absence of fixed pitch landmarks (Weidman and Baker, 2015).

### 1.6 Research gap

Three gaps motivate this study. First, prior research on this dataset and related populations has predominantly reported concurrent associations and group-mean comparisons rather than testing whether self-reported motivational orientation and scheduling pattern retain unique statistical association with specific strategy dimensions once practice quantity, contextual support variables, and the risk of Type I error from multiple testing are addressed. Second, no study of pre-professional string students has modeled these two self-reported correlates as joint, hierarchically entered predictors of five distinct strategy dimensions with complete diagnostic reporting (full coefficients, confidence intervals, and residual diagnostics). Third, no analysis of this population has tested, under an assumption-appropriate model, whether strategic behavior constitutes a statistical mechanism linking motivational orientation to perceived competence and academic achievement, nor has any analysis extended this mediational question to practice-scheduling pattern specifically, despite scheduling being as central to the study’s Research Questions as motivation.

## 2 The present study

### 2.1 Purpose of the study

The purpose of this study is to test whether self-reported motivational orientation (interest/mastery-oriented vs. exam-oriented primary reason for practicing) and self-reported practice-scheduling pattern (daily/structured, unstructured/free-time, or pre-examination) are statistically associated with five dimensions of strategic practice behavior—attention, rehearsal, elaboration, organization, and comprehension monitoring—among pre-professional cello students, after controlling for weekly practice duration and contextual support variables. A second purpose is to test, using an assumption-appropriate model, whether comprehension monitoring statistically mediates the association between both of these self-reported correlates and students’ academic achievement and self-perceived technical-musical competence. Because scheduling pattern is treated symmetrically with motivational orientation throughout the Research Questions and Hypotheses below, the mediation analysis (Section 4.6) tests both variables as focal predictor variables in the mediation model.

### 2.2 Research questions

*RQ1.* Are self-reported motivational orientation and practice-scheduling pattern statistically associated with cello students’ use of each of the five cognitive/metacognitive strategy dimensions?

*RQ2.* Do these associations remain statistically significant, and do they survive correction for multiple comparisons, after weekly practice duration, out-of-school support, and family musical background are statistically controlled?

*RQ3.* Does comprehension-monitoring strategy use statistically mediate the association between (a) self-reported motivational orientation and (b) self-reported practice-scheduling pattern, on the one hand, and students’ academic achievement level and self-perceived technical-musical competence, on the other, under a model specification that does not require the proportional-odds assumption?

### 2.3 Hypotheses

*H1.* Students reporting an interest/mastery-oriented primary reason for practicing will report higher use of elaboration, organizational, and comprehension-monitoring strategies than students reporting an exam-oriented primary reason.

*H2.* Students reporting a daily, structured scheduling pattern will report higher comprehension-monitoring and organizational strategy scores than students reporting unstructured or pre-examination scheduling.

*H3.* In hierarchical regression, self-reported motivational orientation and scheduling pattern will explain a statistically significant increment in variance (Δ*R*^2^) in comprehension-monitoring and elaboration strategy scores beyond weekly practice duration, out-of-school support, and family musical background.

*H4.* Comprehension-monitoring strategy use will statistically mediate the association between (a) self-reported motivational orientation and (b) self-reported practice-scheduling pattern, and academic achievement level and self-perceived technical-musical competence.

## 3 Method

### 3.1 Research design

This study employed a quantitative, cross-sectional predictive-correlational survey design (Fraenkel et al., 2012). This design specifies a directional statistical structure derived from theory—self-reported motivational orientation and scheduling pattern as predictors, strategic behavior as a proximal outcome and hypothesized mediator, and academic achievement/self-perceived competence as distal outcomes—consistent with the forethought-to-performance-to-outcome ordering proposed by cyclical SRL models (Zimmerman, 2002). No experimental manipulation or random assignment was employed. All associations reported below, including those described using predictive or mediational language for consistency with the cited theoretical models, are statistical associations estimated from cross-sectional self-report data and do not, by themselves, establish causal direction; this constraint is revisited throughout the discussion and limitations.

### 3.2 Participants

The study population consisted of cello students enrolled in the cello performance track of Fine Arts High Schools (Güzel Sanatlar Liseleri) affiliated with the Turkish Ministry of National Education. A sample of 166 cello students, recruited through school-level contacts who distributed an anonymous online survey link to eligible students; the total number of students invited was not centrally tracked, and a conventional response rate therefore cannot be computed, was obtained using maximum-variation, non-probability sampling (Creswell, 2012). Because individual school identifiers were not retained in the deidentified dataset made available for this analysis, students’ nesting within schools could not be modeled, and design effects arising from within-school clustering cannot be ruled out; this constraint is discussed in Section 5.7 (Limitations). Table 1 summarizes the demographic and educational characteristics of the sample. The weekly-practice-duration item was collected as unconstrained free text and converted to a numeric hours-per-week value using a documented rule-based procedure: responses stating an explicit weekly total (e.g., “10 saat/hafta”) were used directly; responses stating a daily amount (e.g., “günde 1 saat”) were multiplied by 7 unless the respondent also gave an explicit weekly total, which took precedence; responses giving a numeric range (e.g., “1–2 saat”) were averaged; and responses expressed in minutes were converted to hours. Six responses (3.6% of the sample) could not be converted under any of these rules because they contained no extractable numeric quantity [e.g., “değişiyor, belirli bir saat yok” (“it varies, no fixed amount”)] and were coded as missing. Of the 166 respondents, listwise deletion for these six cases excluded them from regression analyses (*N* = 160); no other study variables contained missing data. A sensitivity analysis comparing listwise deletion with mean-imputation of the missing practice-duration values showed no material change in the pattern or statistical significance of key predictors, supporting the robustness of the reported regression results to this handling of missing data.

**Table 1:** Distribution of participants by demographic and educational characteristics (*N* = 166).

Variable	Category	*f*	%
Gender	Female	109	65.7
	Male	57	34.3
Grade	9th	53	31.9
	10th	40	24.1
	11th	41	24.7
	12th	32	19.3
Attendance	Never absent	101	60.8
	Occasionally absent	65	39.2
Weekly practice duration	Low (0–5 h)	78	47.0
	Moderate (6–10 h)	53	31.9
	High (11 + h)	35	21.1
**Practice-scheduling pattern**	**Daily/structured**	**70**	**42.2**
	**Unstructured/free-time**	**84**	**50.6**
	**Pre-examination**	**12**	**7.2**
Source of strategy acquisition	Teacher	145	87.3
	Self/peers/other	21	12.7
Out-of-school support	Yes	29	17.5
	No	137	82.5
Family musical background	Yes	62	37.3
	No	104	62.7
Cello course achievement (self-reported)	Band 1: 40–60	16	9.6
	Band 2: 61–80	52	31.3
	Band 3: 81–100	98	59.0
Self-perceived competence	Level 1: Low	29	17.5
	Level 2: Moderate	99	59.6
	Level 3: High	38	22.9
**Self-reported primary motivational reason**	**Interest/mastery-oriented**	**136**	**81.9**
	**Exam-oriented**	**30**	**18.1**

The table presents the demographic and educational characteristics of the sample. Percentages may not sum to exactly 100 due to rounding. Bolded rows indicate the two focal self-reported correlates examined in this study.

### 3.3 Instruments

#### 3.3.1 Demographic and contextual information form

An 11-item researcher-developed form collected grade level, gender, attendance, weekly practice duration, self-reported academic achievement band, self-perceived competence, and two focal single-item self-report indicators. The motivation item asked students to select the primary reason they practiced cello, with two response options: “a genuine desire to learn” (coded 1) and “to get by/pass exams” (coded 0); throughout this manuscript these are labeled the *interest/mastery-oriented* and *exam-oriented* primary reasons, respectively, at the level of the item itself, with Self-Determination Theory’s autonomous/controlled distinction invoked only as an interpretive framework rather than as a validated measurement label. The original Turkish item wording was “Viyolonsel dersine çalışmaya motive olduğunuz unşur,” with response options “Tam anlamıyla öğrenme isteği” and “Anı kurtarmak–sınavlardan geçer not almak.” The scheduling item asked students to select their typical practice-scheduling pattern from three options: “every day” (daily/structured), “in free time” (unstructured), or “some time before an exam” (pre-exam); the original Turkish wording was “Viyolonsele çalışma zamanını planlama şekliniz,” with response options “Her gün,” “Serbest zamanda,” and “Sınavdan bir süre önce.” Both items were forced-choice, single-select, and were not drawn from a validated multi-item motivation or self-regulation scale; they are treated throughout this manuscript as coarse, single-item self-report indicators of a broader construct, a measurement limitation discussed in Section 5.7.

#### 3.3.2 Scale of strategies used while practicing and learning instrumental music

Strategic practice behavior was measured with the 39-item self-report scale developed by Aydıner Uygun and Kılınçer (2017), comprising five subdimensions: attention (7 items), rehearsal (5 items), elaboration (6 items), organization (7 items), and comprehension monitoring (14 items), using a five-point Likert-type format (1 = never to 5 = always). In the original validation study, exploratory factor analysis on a sample of Turkish instrumental-music students supported the five-factor structure, with item factor loadings ranging from 0.51 to 0.81, and reported Cronbach’s alpha coefficients of 0.89 (attention), 0.81 (rehearsal), 0.85 (elaboration), 0.87 (organization), and 0.93 (comprehension monitoring) (Aydıner Uygun and Kılınçer, 2017). Internal consistency for the present sample was recalculated (Cronbach’s alpha with bootstrapped 95% confidence intervals) and is supplemented with McDonald’s omega, reported in Section 4.1 (Table 2). Because both the strategy scale and the achievement/competence outcomes were collected via the same self-report instrument at a single time point, common-method variance cannot be ruled out as a partial explanation for the observed associations; this is noted as a limitation in Section 5.7.

**Table 2:** Internal consistency of the strategy scale in the present sample.

Subdimension	*k*	*α* [95% CI]^a^ (present study)	McDonald’s *ω*^b^	*α* (2017 validation)
Attention	7	0.90 [0.87, 0.92]	0.90	0.89
Rehearsal	5	0.82 [0.74, 0.88]	0.83	0.81
Elaboration	6	0.89 [0.85, 0.92]	0.89	0.85
Organization	7	0.83 [0.77, 0.87]	0.83	0.87
Comprehension Monitoring	14	0.90 [0.86, 0.93]	0.91	0.93
Overall scale	39	0.94	—	—

a Bootstrapped 95% confidence interval.

b McDonald’s omega, estimated via iterated principal-axis factoring on each subscale’s items.

All coefficients exceed the 0.70 threshold conventionally regarded as acceptable for group-level research (Nunnally and Bernstein, 1994); omega values closely track alpha, indicating that the internal-consistency estimates are not an artifact of the (essentially) tau-equivalent assumption underlying alpha. The original validation study additionally reported item factor loadings ranging from 0.51 to 0.81 supporting the five-factor structure (Aydıner Uygun and Kılınçer, 2017); the present study did not re-run a confirmatory factor analysis, and this is noted as a limitation in Section 5.7.

### 3.4 Procedure

Data were collected via a self-administered online survey (Google Forms) over an approximately seven-week window, from 6 December 2024 to 28 January 2025, during the 2024–2025 academic year. Participation was voluntary and anonymous, with no course credit or grade incentive offered.

### 3.5 Ethical considerations

This study was conducted in accordance with the Declaration of Helsinki and was approved by the Social and Human Sciences Ethics Committee of Mersin University (Decision No. 83, dated 5 April 2024), obtained prior to data collection. As disclosed in the Acknowledgments, the dataset analyzed here was originally collected for an unpublished master’s thesis authored by one of the co-investigators; the present manuscript’s predictive, mediational research questions, hypotheses, and analytic approach are distinct from that thesis and from a related, descriptively framed manuscript on the same population, and this shared data provenance is disclosed here in the interest of full transparency (see also Acknowledgments).

### 3.6 Data analysis plan

All analyses were conducted in Python 3.12, using pandas 2.x, NumPy 1.26, SciPy 1.11, and statsmodels 0.14 for descriptive, group-comparison, regression, and mediation models; the specific estimator, test, and correction procedure used for each analysis is stated at each step below and, where applicable, in the corresponding Results subsection.

1. *Data screening:* missing values, multivariate outliers (Mahalanobis distance, *p* < 0.001), normality (skewness/kurtosis within ±3; Tabachnick and Fidell, 2013).
2. *Descriptive statistics and reliability:* frequencies, means, SDs; Cronbach’s alpha with bootstrapped 95% CIs and McDonald’s omega for each subscale.
3. *Group comparisons (H1–H2):* Welch’s *t*-test for motivational orientation; Welch’s ANOVA (Field, 2018) (robust to unequal variances) with Games–Howell *post-hoc* comparisons, rather than Tukey HSD, for scheduling pattern, given confirmed variance heterogeneity across groups; Cohen’s *d* and *η*^2^*p* as effect sizes.
4. *Hierarchical multiple regression (H3, RQ1–RQ2):* two-block regression per strategy subdimension, with complete reporting of unstandardized (*B*, SE, 95% CI) and standardized (*β*) coefficients for every predictor in both blocks, full-model *F*-tests, adjusted *R*^2^, and residual diagnostics (Durbin–Watson, Breusch–Pagan, Cook’s distance) for each of the five models.
5. *Mediation analysis (H4, RQ3):* bootstrapped (10,400 resamples; percentile-based confidence intervals) indirect-effect testing (Hayes, 2017) for both self-reported correlates (motivation, scheduling) as focal predictors. Because a preliminary ordinal-logistic specification of the outcome models showed evidence of a violated proportional-odds assumption for the motivation predictor, the primary mediation models reported here instead use multinomial logistic regression (Python 3.12, statsmodels 0.14, MNLogit class, maximum-likelihood estimation via BFGS optimization with warm-started parameters), which imposes no such assumption; results are reported separately for each non-reference outcome category (i.e., each achievement/competence contrast) rather than as a single pooled coefficient. Both scheduling-pattern dummy variables (daily, pre-exam; reference = unstructured) were modeled as separate mediation predictors, so that no category of this three-level variable is omitted from the mediation analysis.
6. *Multiple-comparison correction:* Given that H1, H2, and H3 are each tested across five strategy dimensions, Holm-Bonferroni correction was applied within each hypothesis’s family of five tests; both raw and Holm-adjusted *p*-values are reported, and conclusions are drawn primarily from the corrected values.
7. *Sensitivity and power analyses:* (a) a sensitivity analysis compared listwise deletion (*N* = 160) with mean-imputation (*N* = 166) of missing weekly-practice-duration values in the regression models (Table 3); (b) a *post hoc* sensitivity power analysis (Welch’s *t*-test framework, *α* = 0.05, two-tailed, given the observed *n* = 136/30 group split) established the minimum detectable effect size at 80% power, used to contextualize non-significant findings in Section 4.3.

**Table 3:** Sensitivity analysis: motivation coefficient under listwise deletion vs. mean imputation of missing weekly-hours values.

Strategy	*B* (motivation), listwise deletion *N* = 160	*p*	*B* (motivation), mean-imputed *N* = 166	*p*
Attention	0.010	0.967	0.001	0.997
Rehearsal	0.643	<0.001	0.554	0.003
Elaboration	0.527	0.017	0.478	0.026
Organization	0.258	0.241	0.253	0.242
Comprehension monitoring	0.445	0.007	0.406	0.011

## 4 Results

### 4.1 Preliminary analyses: reliability and data screening

Internal consistency was re-estimated for the present sample and supplemented with McDonald’s omega and bootstrapped 95% confidence intervals for alpha. The results are presented in Table 2.

Six of the 166 free-text responses to the weekly-practice-duration item could not be reliably converted to a numeric value and were coded as missing (listwise *N* = 160 for regression models; see Table 3 for a sensitivity analysis). Skewness and kurtosis values for the five strategy subdimensions ranged from −1.50 to 0.07 and −0.68 to 2.80, respectively, within the ±3 criterion recommended by Tabachnick and Fidell (2013). The results are presented in Table 4, which reports the mean, standard deviation, skewness, kurtosis, and interpretive frequency level for each strategy subdimension.

**Table 4:** Descriptive statistics for strategy subdimensions (*N* = 166).

Strategy dimension	M	SD	Skewness	Kurtosis	Interpretive level*
Attention	3.02	1.09	−0.32	−0.68	Sometimes
Rehearsal	4.11	0.84	−1.50	2.80	Frequently
Elaboration	3.42	0.97	−0.35	−0.22	Frequently
Organization	2.93	0.93	0.07	−0.13	Sometimes
Comprehension monitoring	3.93	0.74	−0.97	1.53	Frequently
Overall Scale	3.53	0.69	−0.61	1.22	Frequently

The table presents descriptive statistics for the five strategy subdimensions. Never (1.00–1.80); rarely (1.81–2.60); sometimes (2.61–3.40); frequently (3.41–4.20); always (4.21–5.00). The * symbol refers to the interpretive-level ranges defined in the table note: Never (1.00–1.80), Rarely (1.81–2.60), Sometimes (2.61–3.40), Frequently (3.41–4.20), Always (4.21–5.00).

### 4.2 Correlations among study variables

To examine bivariate relationships among all study variables, zero-order Pearson correlations were computed for the full set of continuous and dichotomous variables. The results are presented in Table 5, which reports the complete 13 × 13 correlation matrix among weekly practice hours, the two focal self-reported correlates (motivation and, separately, the two scheduling-pattern dummy codes), out-of-school support, family musical background, the five strategy subdimensions, academic achievement, and self-perceived competence—the last of which is now included as an explicit row and column rather than only summarized in the table note. Because several variables are dichotomous, these coefficients are mathematically equivalent to point-biserial correlations; a parallel 13 × 13 Spearman-rank matrix, reported in full in Table 6, produced coefficients within 0.09 of the Pearson values throughout, supporting the use of Pearson/point-biserial correlations as the primary reported statistic.

**Table 5:** Complete zero-order correlation matrix among study variables (*N* = 160–166).

Variable	1	2	3	4	5	6	7	8	9	10	11	12	13
1. Weekly practice hours	—												
2. Motivation (self-reported reason)ᵃ	0.26	—											
3. Daily schedulingᵇ	0.50	0.34	—										
4. Pre-exam schedulingᵇ	−0.22	−0.45	−0.23	—									
5. Out-of-school support	0.30	0.10	0.19	−0.13	—								
6. Family musical background	−0.06	−0.03	−0.02	0.04	0.03	—							
7. Attention	0.18	0.07	0.26	−0.01	0.19	−0.05	—						
8. Rehearsal	0.27	0.38	0.26	−0.20	0.08	−0.04	0.38	—					
9. Elaboration	0.29	0.25	0.18	−0.10	0.17	−0.07	0.46	0.56	—				
10. Organization	0.04	0.09	−0.00	−0.01	0.07	0.09	0.48	0.31	0.52	—			
11. Comprehension monitoring	0.34	0.30	0.25	−0.15	0.17	0.06	0.40	0.69	0.64	0.38	—		
12. Academic achievement	0.35	0.31	0.30	−0.21	0.23	0.02	0.16	0.35	0.28	−0.03	0.33	—	
13. Perceived competence	0.08	0.29	0.13	−0.14	−0.01	−0.02	−0.03	0.22	0.16	−0.04	0.14	0.40	—

Coefficients ≥ |0.16| are significant at *p* < 0.05 and coefficients ≥ |0.20| at *p* < 0.01 for this sample size (two-tailed, *N* = 160–166). Motivation and daily scheduling were themselves correlated (*r* = 0.34), consistent with their being related but statistically distinguishable correlates, as confirmed by the variance inflation factors reported in Table 10 (all VIFs < 2.6). A full 13 × 13 Spearman-rank correlation matrix, computed as a robustness check given the dichotomous coding of several variables, is reported in Table 6; the maximum absolute difference between corresponding Pearson and Spearman coefficients was 0.09, supporting the use of Pearson/point-biserial coefficients as the primary reported statistic in Table 5. ^a^ single-item, dichotomously coded (0/1) self-reported variable; ^b^ dummy-coded contrast of the three-level scheduling-pattern variable (reference category = Unstructured/free-time, not shown as a separate row).

**Table 6:** Complete 13 × 13 Spearman-Rank correlation matrix (robustness check for Table 5).

Variable	1	2	3	4	5	6	7	8	9	10	11	12	13
1. Weekly practice hours	—												
2. Motivation (self-reported reason)	0.33	—											
3. Daily scheduling	0.55	0.34	—										
4. Pre-exam scheduling	−0.31	−0.45	−0.23	—									
5. Out-of-school support	0.21	0.10	0.19	−0.13	—								
6. Family musical background	−0.01	−0.03	−0.02	0.04	0.03	—							
7. Attention	0.23	0.09	0.28	−0.03	0.18	−0.06	—						
8. Rehearsal	0.32	0.33	0.26	−0.17	0.07	0.01	0.39	—					
9. Elaboration	0.33	0.23	0.18	−0.09	0.18	−0.05	0.45	0.53	—				
10. Organization	0.06	0.08	0.02	−0.00	0.07	0.08	0.47	0.29	0.48	—			
11. Comprehension Monitoring	0.36	0.30	0.22	−0.11	0.19	0.08	0.39	0.64	0.64	0.35	—		
12. Academic achievement	0.42	0.27	0.31	−0.17	0.25	0.02	0.16	0.28	0.26	−0.06	0.33	—	
13. Perceived competence	0.13	0.29	0.13	−0.14	−0.01	−0.02	−0.03	0.21	0.14	−0.08	0.13	0.40	—

The table reports the complete Spearman-rank correlation matrix corresponding to Table 5. Coefficients are within 0.09 of their Pearson/point-biserial counterparts throughout (maximum absolute difference = 0.090, for the Attention–Pre-exam-scheduling pair), supporting the use of Pearson/point-biserial coefficients as the primary reported statistic in Table 5.

### 4.3 Test of hypothesis 1: motivational orientation and strategy use

To test Hypothesis 1, Welch’s *t*-tests (which do not assume equal variances) compared strategy subdimension scores between students reporting an interest/mastery-oriented primary reason (*n* = 136) and an exam-oriented primary reason (*n* = 30). Because five dimensions were tested, Holm–Bonferroni correction was applied within this family of five tests; both raw and corrected *p*-values are reported. A *post hoc* sensitivity power analysis indicated that, given this group split, the study had 80% power to detect effects of *d* ≥ 0.57; observed power for the non-significant Attention and Organization effects (*d* = 0.22 and 0.23) was only 0.19 and 0.21, respectively, so these null results should be interpreted as inconclusive rather than as evidence of no association. The results are presented in Table 7.

**Table 7:** Independent-samples comparison of strategy use by motivational orientation, with Holm-Adjusted *p*-values and achieved power.

Strategy	Interest/mastery-oriented (*n* = 136) M (SD)	Exam-oriented (*n* = 30) M (SD)	*t*	df	*p* (raw)	*p* (Holm)	Cohen’s *d*	Achieved power^a^
Attention	3.06 (1.09)	2.83 (1.07)	1.08	40.9	0.285	0.462	0.22	0.19
Rehearsal	4.24 (0.73)	3.51 (1.04)	3.70	36.9	<0.001	0.004	0.93	0.996
Elaboration	3.53 (0.93)	2.93 (1.00)	2.99	39.9	0.005	0.014	0.63	0.874
Organization	2.97 (0.94)	2.76 (0.84)	1.21	43.0	0.231	0.462	0.23	0.21
Comprehension monitoring	4.03 (0.70)	3.48 (0.77)	3.61	40.4	<0.001	0.004	0.77	0.967

H1 is supported for rehearsal, elaboration, and comprehension monitoring after Holm–Bonferroni correction (all *p*_Holm ≤ 0.014), and is not supported for attention or organization, although the latter two tests were underpowered (achieved power 0.19–0.21 for the observed effect sizes) rather than clearly null.

a Achieved power computed *post hoc* for the observed effect size, given *n* = 136/30, *α* = 0.05, two-tailed.

### 4.4 Test of hypothesis 2: practice-scheduling pattern and strategy use

Because Levene’s test indicated significant variance heterogeneity across the three scheduling groups for several subdimensions, Welch’s ANOVA (robust to unequal variances) was used in place of the standard *F*-test, and Games–Howell *post-hoc* comparisons (which do not assume equal variances or equal sample sizes) were used in place of Tukey’s HSD. Holm–Bonferroni correction was applied within this family of five omnibus tests. The results are presented in Table 8.

**Table 8:** Welch’s ANOVA: strategy use by practice-scheduling pattern, with Holm-adjusted *p*-values.

Strategy	Daily M (SD)	Unstruct. M (SD)	Pre-exam M (SD)	Welch *F* (df1, df2)	*p*	*p* (Holm)	*η* ^2^ *p*
Attention	3.38 (1.10)	2.74 (1.02)	2.92(1.09)	7.18 (2,30.7)	0.003	0.012	0.080
Rehearsal	4.38 (0.63)	3.96 (0.91)	3.55 (1.09)	8.13 (2,28.8)	0.002	0.010	0.094
Elaboration	3.64 (0.90)	3.28 (1.00)	3.11 (1.04)	3.41 (2,30.2)	0.046	0.092	0.039
Organization	2.95 (0.93)	2.92 (0.93)	2.88 (0.96)	0.04 (2,30.7)	0.961	0.961	0.001
Comprehension monitoring	4.17 (0.63)	3.78 (0.77)	3.57 (0.85)	7.39 (2,28.7)	0.003	0.012	0.080

The table presents Welch’s ANOVA (robust to unequal variances) for strategy use by scheduling pattern. After Holm correction, the omnibus test remains significant for attention, rehearsal, and comprehension monitoring (all *p*_Holm ≤ 0.012), but the elaboration effect (raw *p* = 0.046) does not survive correction (*p*_Holm = 0.092) and should be treated as non-significant. Organization was not significant (*p* = 0.961).

Because the Welch’s ANOVA results in Table 8 indicated statistically significant omnibus differences for Attention, Rehearsal, and Comprehension Monitoring, Games–Howell pairwise comparisons were conducted to identify which specific scheduling-pattern groups differed on these three dimensions. The results are presented in Table 9, which reports the mean difference, standard error, *t*-statistic, degrees of freedom, and *p*-value for each statistically significant pairwise contrast.

**Table 9:** Games–Howell *post-hoc* comparisons for omnibus-significant strategy dimensions.

Strategy	Comparison	Mean diff.	SE	*t* (df)	*p* (Games–Howell)
Attention	Daily > unstructured	0.64	0.17	3.80 (150.9)	<0.001
Rehearsal	Daily > unstructured	0.42	0.12	3.48 (144.5)	0.001
	Daily > pre-exam	0.83	0.33	2.55 (12.1)	0.025
Comprehension monitoring	Daily > unstructured	0.38	0.11	3.57 (145.3)	0.001
	Daily > pre-exam	0.59	0.30	1.96 (12.0)	0.073 (ns)

H2 is supported for comprehension monitoring with respect to the daily-vs-unstructured contrast (*p* = 0.001), but the daily-vs-pre-exam contrast for comprehension monitoring, which had appeared significant under the previously reported Tukey HSD procedure (*p* = 0.023), is not statistically significant under the variance-robust Games–Howell procedure (*p* = 0.073) and is now reported as non-significant; this revision reflects the more conservative and appropriate *post-hoc* method given confirmed variance heterogeneity. H2 is not supported for organization (*F* = 0.04, *p* = 0.961), the dimension whose label most closely resembles “scheduling” itself. Significant, unhypothesized differences also emerged for attention and rehearsal.

### 4.5 Test of hypothesis 3: hierarchical regression analyses

For each of the five strategy subdimensions, weekly practice duration (standardized), out-of-school support, and family musical background were entered in Block 1; self-reported motivational orientation and two scheduling-pattern dummy codes (reference = Unstructured) were entered in Block 2. Listwise *N* = 160. Full, complete-case coefficients for every predictor in both blocks, together with residual diagnostics, are reported below to allow full evaluation of model specification and multicollinearity. The results are presented in Tables 10–15.

**Table 10:** Model summary and residual diagnostics for the five hierarchical regression models.

Criterion	*R*^2^₁	*R*^2^₂	Adj. *R*^2^₂	Δ*R*^2^ (*p*)	Full model *F* (*p*)	Durbin–Watson	Breusch–Pagan *p*
Attention	0.054	0.094	0.059	0.040 (0.086)	2.65 (0.018)	1.76	0.838
Rehearsal	0.072	0.179	0.147	**0.107 (<0.001)**	5.57 (<0.001)	1.93	**0.046**
Elaboration	0.093	0.129	0.095	0.036 (0.104)	3.78 (0.002)	1.97	0.402
Organization	0.012	0.022	−0.017	0.010 (0.684)	0.56 (0.761)	1.83	0.386
Monitoring	0.124	0.177	0.145	**0.053 (0.022)**	5.50 (<0.001)	2.04	0.080

Durbin–Watson values near 2 indicate no evidence of autocorrelated residuals. Breusch–Pagan *p* = 0.046 for the rehearsal model indicates statistically significant heteroscedasticity; HC3 heteroscedasticity-consistent standard errors, confidence intervals, and *p*-values are therefore reported as the primary inference for this model (Table 11), rather than the conventional OLS standard errors used for the other four models, which showed no evidence of heteroscedasticity (all Breusch–Pagan *p* > 0.08). Maximum Cook’s distance across all five models was 0.09, well below the conventional concern threshold of 1, indicating no unduly influential cases.

**Table 11:** Complete hierarchical regression coefficients—attention.

Predictor	*B*	SE	95% CI	*β*	*t*	*p*
Weekly practice hours (*z*)	0.035	0.099	[−0.16, 0.23]	0.033	0.35	0.725
Out-of-school support	0.411	0.225	[−0.03, 0.85]	0.148	1.83	0.069
Family musical background	−0.103	0.170	[−0.44, 0.23]	−0.047	−0.61	0.546
Motivation (interest/mastery-oriented = 1)	0.010	0.249	[−0.48, 0.50]	0.004	0.04	0.967
Daily scheduling (dummy)	**0.492**	0.199	[0.10, 0.89]	0.228	2.47	**0.015**
Pre-exam scheduling (dummy)	0.304	0.368	[−0.42, 1.03]	0.072	0.83	0.411

**Table 12:** Complete hierarchical regression coefficients—rehearsal.

Predictor	*B*	SE (HC3)	95% CI (HC3)	*β*	*t* (HC3)	*p* (HC3)
Weekly practice hours (*z*)	**0.119**	**0.059**	**[0.00, 0.24]**	**0.147**	**2.02**	**0.043**
Out-of-school support	−0.027	0.138	[−0.30, 0.24]	−0.013	−0.19	0.847
Family musical background	−0.028	0.130	[−0.28, 0.23]	−0.017	−0.21	0.831
Motivation (interest/mastery-oriented = 1)	**0.643**	**0.229**	**[0.19, 1.09]**	**0.307**	**2.81**	**0.005**
Daily scheduling (dummy)	0.137	0.132	[−0.12, 0.40]	0.084	1.04	0.300
Pre-exam scheduling (dummy)	−0.038	0.396	[−0.81, 0.74]	−0.012	−0.10	0.924

**Table 13:** Complete hierarchical regression coefficients—elaboration.

Predictor	*B*	SE	95% CI	*β*	*t*	*p*
Weekly practice hours (z)	**0.207**	0.087	[0.04, 0.38]	0.216	2.38	**0.018**
Out-of-school support	0.233	0.196	[−0.15, 0.62]	0.094	1.19	0.237
Family musical background	−0.104	0.149	[−0.40, 0.19]	−0.053	−0.70	0.485
Motivation (interest/mastery-oriented = 1)	**0.527**	0.217	[0.10, 0.96]	0.213	2.43	**0.017**
Daily scheduling (dummy)	−0.002	0.174	[−0.35, 0.34]	−0.001	−0.01	0.991
Pre-exam scheduling (dummy)	0.232	0.322	[−0.40, 0.87]	0.062	0.72	0.472

**Table 14:** Complete hierarchical regression coefficients—comprehension monitoring.

Predictor	*B*	SE	95% CI	*β*	*t*	*p*
Weekly practice hours (*z*)	**0.177**	0.065	[0.05, 0.30]	0.242	2.72	**0.007**
Out-of-school support	0.133	0.146	[−0.15, 0.42]	0.070	0.91	0.363
Family musical background	0.118	0.111	[−0.10, 0.34]	0.079	1.06	0.286
Motivation (interest/mastery-oriented = 1)	**0.445**	0.162	[0.13, 0.76]	0.235	2.75	**0.007**
Daily scheduling (dummy)	0.069	0.130	[−0.19, 0.33]	0.047	0.53	0.593
Pre-exam scheduling (dummy)	0.079	0.239	[−0.39, 0.55]	0.027	0.33	0.741

**Table 15:** Complete hierarchical regression coefficients—organization.

Predictor	*B*	SE	95% CI	*β*	*t*	*p*
Weekly practice hours (z)	0.028	0.087	[−0.14, 0.20]	0.031	0.32	0.748
Out-of-school support	0.138	0.198	[−0.25, 0.53]	0.058	0.70	0.488
Family musical background	0.162	0.150	[−0.13, 0.46]	0.087	1.08	0.282
Motivation (interest/mastery-oriented = 1)	0.258	0.219	[−0.17, 0.69]	0.110	1.18	0.241
Daily scheduling (dummy)	−0.100	0.176	[−0.45, 0.25]	−0.054	−0.57	0.570
Pre-exam scheduling (dummy)	0.135	0.324	[−0.51, 0.78]	0.038	0.42	0.677

The results of the five hierarchical regression models are presented in Tables 10–15. Table 10 reports, for each strategy dimension, the variance explained at Block 1 and Block 2 (*R*^2^₁, *R*^2^₂), adjusted *R*^2^, the increment in variance associated with Block 2 (Δ*R*^2^) with its significance test, the full-model *F*-test, and three residual diagnostics: the Durbin–Watson statistic (independence of residuals), the Breusch–Pagan test (homoscedasticity), and the maximum Cook’s distance across cases (influential-case screening). Variance inflation factors for the six Block 2 predictors, computed once since the predictor set is identical across all five models, ranged from 1.09 to 2.55, well below the threshold of 5 conventionally used to flag problematic multicollinearity.

Table 11 reports the complete set of Block 1 and Block 2 coefficients for Attention. Neither block contributed statistically significant unique variance from motivational orientation, but the daily-scheduling predictor was a significant unique correlate (*B* = 0.492, *p* = 0.015), the only strategy dimension for which scheduling, rather than motivation, was the significant Block 2 predictor.

Table 12 reports the complete set of Block 1 and Block 2 coefficients for rehearsal. Because the Breusch–Pagan test indicated statistically significant heteroscedasticity for this model (*p* = 0.046), HC3-robust standard errors, confidence intervals, and *p*-values are reported as the primary inference. Motivational orientation was the only statistically significant Block 2 predictor under HC3 inference [*B* = 0.643, SE_h_ᴄ₃ = 0.229, 95% CI (0.195, 1.090), *p* = 0.005], consistent with the large effect size observed for this dimension in Table 7; weekly practice hours, not significant under conventional OLS inference (*p* = 0.096), reached significance under HC3 inference [SE_h_ᴄ₃ = 0.059, 95% CI (0.004, 0.235), *p* = 0.043].

Table 13 reports the complete set of Block 1 and Block 2 coefficients for elaboration. Motivational orientation remained a statistically significant unique correlate (*B* = 0.527, *p* = 0.017) despite the non-significant omnibus Δ*R*^2^ test reported in Table 10, because the scheduling-pattern dummy variables contributed negligible additional unique variance in this model.

Table 15 reports the complete set of Block 1 and Block 2 coefficients for organization. No predictor in either block reached statistical significance, consistent with the non-significant omnibus Δ*R*^2^ test in Table 10 and the non-significant Welch’s ANOVA result in Table 8 for this dimension.

Table 14 reports the complete set of Block 1 and Block 2 coefficients for comprehension Monitoring. Both weekly practice hours (*B* = 0.177, *p* = 0.007) and motivational orientation (*B* = 0.445, *p* = 0.007) were statistically significant unique correlates in the full model.

Tables 11–15 present unstandardized (*B*, SE, 95% CI) and standardized (*β*) coefficients for every Block 1 and Block 2 predictor (Table 12 reports HC3-robust values as primary); bold values are significant at *p* < 0.05. Holm–Bonferroni correction was applied to the family of five block-level Δ*R*^2^ *F*-change tests (one per strategy dimension); individual regression-coefficient *p*-values within each model are reported uncorrected, as descriptive of model structure rather than as independently multiplicity-corrected inferences. H3, as prespecified, hypothesized a significant Δ*R*^2^ for elaboration and comprehension monitoring specifically. Neither survives Holm correction (comprehension monitoring: Δ*R*^2^ = 0.053, *p*_Holm = 0.089; elaboration: Δ*R*^2^ = 0.036, *p*_Holm = 0.258), so *H3 is not supported as prespecified*. The only strategy dimension whose Δ*R*^2^ survives Holm correction is rehearsal (Δ*R*^2^ = 0.107, *p*_Holm = 0.0015)—a dimension H3 did not specify—and this is reported as an *unanticipated, exploratory finding* rather than as support for H3. Notwithstanding the non-significant omnibus Δ*R*^2^ test for Elaboration, the individual motivation coefficient in that model remains nominally significant (*B* = 0.527, *p* = 0.017, uncorrected); because this coefficient is not part of the Holm-corrected family, it should be read descriptively rather than as confirmatory evidence. For Attention, scheduling (not motivation) is the significant unique predictor (*B* = 0.492, *p* = 0.015, uncorrected), a reversal of the pattern hypothesized for this dimension. A sensitivity analysis (Table 3) comparing listwise deletion (*N* = 160) with mean-imputed weekly-hours values (*N* = 166) showed materially unchanged coefficients and significance patterns for the motivation predictor across all five models, supporting the robustness of these results to the handling of missing practice-duration data.

### 4.6 Test of hypothesis 4: mediation analysis

Comprehension-monitoring strategy use was tested as a statistical mediator between each of the three self-reported focal predictors (motivational orientation, daily scheduling, and pre-exam scheduling) and (a) academic achievement and (b) self-perceived competence, controlling for weekly practice hours, out-of-school support, family musical background, and, reciprocally, the other two focal predictors. Because a preliminary diagnostic check indicated that the proportional-odds assumption did not hold for the association between motivational orientation and academic achievement (coefficients of 1.81 and 0.35 at the two cumulative cutoffs), the mediation models were estimated using multinomial logistic regression, which imposes no such assumption, for both outcome variables. The indirect effect for all six pathways was bootstrapped jointly across 10,400 resamples (nonparametric case resampling with replacement; percentile-based 95% confidence intervals; random seeds 4,000–4,008, run in nine chunks of 800–1,200 resamples each for computational tractability, using Python 3.12 and statsmodels 0.14), so that the path-b coefficients (the association between comprehension monitoring and each outcome) are estimated from a single, consistent model containing all three focal predictors simultaneously and are therefore identical across the three predictor-specific pathways for a given outcome and category contrast; only the path-a coefficient (the association between each focal predictor and comprehension monitoring, controlling for the other two) differs across pathways. Because multinomial models estimate one coefficient per non-reference outcome category, each pathway below is reported as two indirect effects: a Band-2-vs-1 (or Level-2-vs-1) contrast and a Band-3-vs-1 (or Level-3-vs-1) contrast, yielding twelve indirect effects in total (three focal predictors × two outcomes × two category contrasts).

## 5 Discussion

### 5.1 Overview

This study examined whether self-reported motivational orientation and practice-scheduling pattern are statistically associated with five dimensions of strategic practice behavior among pre-professional cello students, and whether comprehension monitoring statistically mediates the association between these correlates and academic achievement and self-perceived competence. Motivational orientation was associated with rehearsal, elaboration, and comprehension-monitoring strategies, robust to Holm-Bonferroni correction for rehearsal and comprehension monitoring specifically; scheduling pattern was associated with comprehension monitoring but not organizational strategy use; and, once the mediation model was re-specified to satisfy statistical assumptions, no mediating role of comprehension monitoring was supported for either correlate or either outcome. These results are considered below in relation to Self-Determination Theory (SDT), self-regulated learning (SRL) theory, and relevant prior work on self-regulated instrumental practice (López-Íñiguez and McPherson, 2020; Tao et al., 2024).

### 5.2 Motivational orientation and scheduling pattern as correlates of strategic behavior (H1–H3)

Consistent with SDT (Deci and Ryan, 2000; Ryan and Deci, 2020), self-reported interest/mastery-oriented motivation was associated with elaboration and comprehension-monitoring strategy use at the bivariate level (Table 7), surviving Holm correction within that family of tests for comprehension monitoring specifically (Table 7). However, once weekly practice duration and scheduling pattern were entered as covariates in the hierarchical regression models, neither the Elaboration nor the Comprehension Monitoring block-level association survived Holm correction (Tables 10–15); H3, which had specifically hypothesized these two dimensions, is therefore not supported as prespecified. Instead, the regression finding that did survive correction was an unhypothesized, exploratory association between motivation and rehearsal (*d* = 0.93 at the bivariate level; Δ*R*^2^ = 0.107, *p*_Holm = 0.0015 in the regression model), the largest effect observed anywhere in this study. This departed from the assumption that repetition-based strategies are motivationally inert (Vansteenkiste et al., 2006); we report it explicitly as a descriptively striking, hypothesis-generating pattern rather than a confirmed mechanism, since a plausible account—for example, that self-determined engagement is expressed behaviorally through sustained, concentrated repetition, consistent with Ericsson et al.’s (1993) construct of deliberate practice—cannot be distinguished from reverse or reciprocal association in this cross-sectional design, and we recommend this specific association be treated as a candidate for pre-registered replication rather than as an established finding of this study.

Scheduling pattern showed a more selective pattern of association, robust for comprehension monitoring (surviving Holm correction) but entirely absent for organizational strategy use (Δ*R*^2^ = 0.010, *p* = 0.684; Welch *F* = 0.04, *p* = 0.961)—the one strategy dimension whose label most closely resembles “scheduling.” This null result, replicated across two independent analytic approaches (ANOVA and regression), suggests that within-session organizational technique (grouping notational or rhythmic material) and between-session scheduling regularity are empirically distinct components of self-regulation that should not be conflated in future instrumental-SRL research, possibly because organizational technique is taught directly by studio teachers (87.3% of this sample reported the teacher as their primary strategy source) regardless of a student’s own scheduling habits.

### 5.3 The absence of a statistically supported mediation mechanism (H4)

The mediation analysis is the result requiring the most careful interpretation in this study. Because a preliminary diagnostic check indicated that the proportional-odds assumption did not hold for the association between motivational orientation and academic achievement (with markedly different coefficients across cumulative cutoffs), the mediation models were estimated using multinomial logistic regression, which imposes no such assumption, with the indirect effect for all three focal predictors bootstrapped jointly across 10,400 resamples to maximize estimation precision. None of the twelve resulting indirect effects (three self-reported focal predictors × two outcomes × two category contrasts) reached statistical significance, indicating that comprehension monitoring does not function as a statistically detectable mediator of any of these associations in this sample. This pattern illustrates a broader methodological point relevant to this literature: ordinal outcome models used in mediation analysis should be routinely checked for proportional-odds violations, since such violations can produce misleading coefficient estimates that do not reflect a stable, assumption-appropriate effect.

The absence of a statistically supported mediation pathway does not imply that motivation and comprehension monitoring are unrelated to achievement and competence; motivation and monitoring were each independently associated with several strategy and outcome variables in the correlation matrix (Table 5) and regression models (Tables 7, 10–15). Rather, the specific indirect pathway through comprehension monitoring, as operationalized and tested here, is not statistically supported once appropriate model assumptions are satisfied and estimation precision is maximized. This is consistent with a broader pattern in which self-perceptions of competence and single-timepoint achievement bands may be shaped by a wider range of affective, developmental, and pedagogical inputs than a single cognitive strategy variable can capture (López-Íñiguez and McPherson, 2021; Tao et al., 2024).

### 5.4 Limitations

#### 5.4.1 Measurement

Both focal correlates (motivational orientation, scheduling pattern) were assessed with single forced-choice items rather than validated multi-item scales, collapsing constructs that SDT and SRL theory treat as continua; this coarse operationalization likely attenuates observed associations and cannot capture within-category heterogeneity or motivational change over time. The strategy scale and the achievement/competence outcomes were collected via the same self-report instrument at a single time point, raising the possibility of common-method variance. No confirmatory factor analysis was conducted on the strategy scale in the present sample; reliance is placed on the original validation study’s exploratory factor structure (loadings 0.51–0.81; Aydıner Uygun and Kılınçer, 2017).

#### 5.4.2 Sampling and clustering

The non-probability, maximum-variation sample does not support formal statistical generalization to the national population of pre-professional cello students, and findings should be read as characterizing this sample. Because individual school identifiers were not retained in the deidentified dataset, students’ nesting within an estimated 26 schools could not be modeled, and standard errors reported throughout (including in Tables 7–16) do not account for potential within-school clustering; to the extent that practice culture or teacher effects are shared within schools, these design effects could inflate apparent precision, and this constraint should be weighed when interpreting borderline *p*-values. A conventional response rate could not be computed because the number of students invited was not centrally tracked.

**Table 16:** Multinomial-model mediation analysis: motivational orientation and both scheduling-pattern contrasts (daily, pre-exam) via comprehension monitoring.

Pathway	Contrast	*a*	*b*	*a* × *b* (point)	95% bootstrap CI (10,400 resamples)
Motivation → monitoring → achievement	Band 2 vs. 1	0.445	0.0094	0.0042	[−0.395, 0.686]
	Band 3 vs. 1	0.445	0.5199	0.2311	[−0.102, 1.108]
Motivation → monitoring → competence	Level 2 vs. 1	0.445	−0.1033	−0.0459	[−0.307, 0.356]
	Level 3 vs. 1	0.445	0.1940	0.0863	[−0.195, 0.657]
Daily scheduling → monitoring → achievement	Band 2 vs. 1	0.069	0.0094	0.0007	[−0.149, 0.214]
	Band 3 vs. 1	0.069	0.5199	0.0361	[−0.224, 0.328]
Daily scheduling → monitoring → competence	Level 2 vs. 1	0.069	−0.1033	−0.0072	[−0.121, 0.088]
	Level 3 vs. 1	0.069	0.1940	0.0135	[−0.128, 0.211]
Pre-exam scheduling → monitoring → achievement	Band 2 vs. 1	0.079	0.0094	0.0007	[−0.492, 0.409]
	Band 3 vs. 1	0.079	0.5199	0.0412	[−0.661, 0.714]
Pre-exam scheduling → monitoring → competence	Level 2 vs. 1	0.079	−0.1033	−0.0082	[−0.332, 0.169]
	Level 3 vs. 1	0.079	0.1940	0.0154	[−0.407, 0.435]

H4 is not supported for any of the three self-reported focal predictors, either outcome, or either category contrast: all twelve 95% bootstrap confidence intervals for the indirect effect (*a* × *b*) include zero, indicating that comprehension monitoring does not function as a statistically detectable mediator of any of these associations in this sample. Because path *b* (the monitoring-to-outcome coefficient) is estimated from a single model containing all three focal predictors, its value is identical across the three predictor-specific rows for a given outcome/contrast; only path a and, consequently, the indirect effect *a* × *b* differ across rows.

#### 5.4.3 Multiple comparisons and power

With five strategy dimensions tested under each of three hypotheses, Holm–Bonferroni correction was applied throughout; several findings that were significant at the uncorrected level (the elaboration omnibus ANOVA, the Comprehension-Monitoring Δ*R*^2^ test, and one Games–Howell contrast) did not survive this correction and are reported as non-significant. The study additionally had limited power (as low as 0.19–0.21) to detect the small-to-moderate effects observed for attention and organization, so null findings for these dimensions should be read as inconclusive.

#### 5.4.4 Design

The cross-sectional, self-report design does not support causal inference; directional language used elsewhere in this manuscript reflects *a priori* theoretical ordering, not confirmed causal sequence. Reverse or reciprocal association (e.g., strategy use shaping self-reported motivation) cannot be ruled out.

#### 5.4.5 Secondary data

As disclosed in the Method and Acknowledgments, this dataset was originally collected for an unpublished master’s thesis and has also informed a separate, descriptively framed manuscript on the same population; while the present predictive/mediational framing is original, this shared provenance constrains the independence of evidence across these outputs.

### 5.5 Recommendations for future research

Future work should (a) replace the single-item motivation and scheduling measures with validated multi-item scales; (b) record school identifiers to permit multilevel modeling of within-school clustering; (c) employ longitudinal or experimental designs capable of adjudicating directionality, particularly for the unexpected motivation–rehearsal association; (d) pre-register mediation hypotheses and routinely check proportional-odds assumptions before reporting ordinal-outcome mediation effects; and (e) incorporate emotion-regulation measures, which prior single-case work suggests may explain self-perceived competence better than cognitive strategy variables alone (López-Íñiguez and McPherson, 2021).

## 6 Conclusion

This study examined whether self-reported motivational orientation and practice-scheduling pattern are statistically associated with five dimensions of self-regulated strategic behavior among 166 pre-professional cello students, and whether comprehension monitoring mediates the association between these correlates and academic achievement and self-perceived competence. Self-reported motivational orientation was associated with several strategy dimensions at the bivariate level, but after Holm–Bonferroni correction and control for practice quantity and scheduling pattern, the hypothesized (H3) associations with elaboration and comprehension monitoring were not supported as prespecified; instead, the only regression association to survive correction was an unhypothesized, exploratory one with rehearsal, which we report as hypothesis-generating rather than confirmatory. Scheduling pattern was associated with comprehension monitoring but not organizational strategy use, an unanticipated dissociation suggesting these are empirically distinct self-regulation components. No statistically supported mediation pathway through comprehension monitoring was found for either self-reported correlate, either outcome, or any of the scheduling-pattern contrasts, under an assumption-appropriate multinomial specification; this null result is this study’s substantive conclusion regarding the proposed mediating mechanism. These findings should be read as correlational, based on coarse self-report measures and a non-probability, single-country sample without school-level clustering information, and are offered as hypothesis-generating evidence for a research literature that would benefit from more assumption-checked and pre-registered mediation testing than has typically been reported to date.

## Data Availability

The original contributions presented in the study are included in the article/supplementary material, further inquiries can be directed to the corresponding author.
